# Infernal 1.1: 100-fold faster RNA homology searches

**DOI:** 10.1093/bioinformatics/btt509

**Published:** 2013-09-04

**Authors:** Eric P. Nawrocki, Sean R. Eddy

**Affiliations:** HHMI Janelia Farm Research Campus, Ashburn, VA 20147, USA

## Abstract

**Summary:** Infernal builds probabilistic profiles of the sequence and secondary structure of an RNA family called covariance models (CMs) from structurally annotated multiple sequence alignments given as input. Infernal uses CMs to search for new family members in sequence databases and to create potentially large multiple sequence alignments. Version 1.1 of Infernal introduces a new filter pipeline for RNA homology search based on accelerated profile hidden Markov model (HMM) methods and HMM-banded CM alignment methods. This enables ∼100-fold acceleration over the previous version and ∼10 000-fold acceleration over exhaustive non-filtered CM searches.

**Availability:** Source code, documentation and the benchmark are downloadable from http://infernal.janelia.org. Infernal is freely licensed under the GNU GPLv3 and should be portable to any POSIX-compliant operating system, including Linux and Mac OS/X. Documentation includes a user’s guide with a tutorial, a discussion of file formats and user options and additional details on methods implemented in the software.

**Contact:** nawrockie@janelia.hhmi.org

## 1 INTRODUCTION

Many structural RNAs conserve their sequence and secondary structure, and the most effective RNA homology search and alignment tools incorporate both types of conservation into their scoring systems. Covariance models (CMs) are profile stochastic context-free grammars (Durbin *et al.*, 1998), probabilistic models of the conserved sequence and secondary structure of an RNA family, analogous to sequence-based profile hidden Markov models (HMMs) commonly used for protein sequence analysis, with added complexity necessary for modeling RNA secondary structure. Infernal implements methods for constructing CMs from input structurally annotated RNA alignments or single sequences and for using those models to search for and align homologous RNAs.

**Fig. 1.**
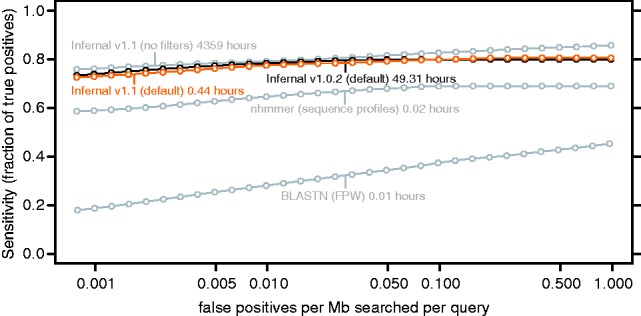
ROC-like curves for the benchmark. Plots are shown for the new Infernal 1.1 with and without filters, for the old Infernal 1.0.2, for profile HMM searches with nhmmer (from the HMMER package included in Infernal 1.1, default parameters) and for family-pairwise-searches with BLASTN (ncbi-blast-2.2.28+, default parameters). The maximum sensitivity (not shown) for default Infernal 1.1 is 0.81 (629 of 820 true positives found), which is achieved at a false-positive rate of 0.19/Mb/query. For non-filtered Infernal, maximum sensitivity is 0.87 at 2.9 false positives per Mb per query. This indicates that at high false-positive rates the filters prevent some true positives from being found, but prevent many more false positives from being found. CPU times are total times for all 106 family searches measured for single execution threads on 3.0 GHz Intel Xeon processors. The Infernal times do not include time required for model calibration.

Compared with the previous version 1.0.2, Infernal 1.1 accelerates typical RNA homology searches ∼100-fold using a filter pipeline based on accelerated profile HMM methods [the HMMER3 project (Eddy, 2008, 2011)] and constrained CM alignment algorithms (Brown, 2000; Nawrocki, 2009). The increased speed comes at a negligible cost to sensitivity (Fig. 1). Additionally, version 1.1 implements specialized algorithms for structural alignment of truncated RNA sequences (Kolbe and Eddy, 2009) commonly found in sequencing reads, which were prone to misalignment in previous versions.

## 2 APPROACH

Exhaustive dynamic programming (DP) CM algorithms are impractically slow (Fig. 1). Several types of sequence-based filters have been developed for acceleration, including a BLAST-based filtering scheme used by Rfam since its inception (Griffiths-Jones *et al.*, 2003) and several profile HMM-based methods (Weinberg and Ruzzo, 2004, 2006). Infernal version 1.0.2 and version 1.1 both use profile HMM filters: version 1.0.2’s filters are derived from the HMMER2 package (Eddy, 2003), whereas version 1.1 co-opts HMMER3’s dramatically accelerated search algorithms, which take advantage of single-instruction multiple-data vector instructions to parallelize the core steps of the HMM DP algorithms (Eddy, 2011). Version 1.1 uses four separate profile HMM-based filter stages, each one successively slower and stricter than the previous stage. The new filter stages are sufficiently fast that the post-HMM-filtering CM DP algorithms as implemented in the previous version (1.0.2) became the clear computational bottleneck. To accelerate these, constraints, or bands, derived from an HMM alignment of the sequence are imposed on the DP matrices to significantly reduce the number of required calculations (Brown, 2000; Nawrocki, 2009). Both the new filters and the banded CM methods are vital for the improved search speed. In the benchmark described later in the text, for default Infernal searches, the profile HMM stages take about one-third of the total running time and the remaining time is spent on the subsequent CM DP calculations.

## 3 USAGE

There are two major applications of Infernal: to search for structural RNAs in a sequence dataset (e.g. to perform genome annotation of RNAs) and to create multiple sequence- and structure-based alignments of RNA homologs [e.g. 16S small subunit ribosomal RNA alignment for environmental survey studies (Cole *et al.*, 2009)]. Both applications begin with a CM file, which can either be downloaded from the Rfam database of >2000 RNA families (Burge *et al.*, 2013) or created by the user with Infernal’s *cmbuild* program from a structurally annotated single sequence or multiple sequence alignment. Before a CM can be used to search a sequence database, it must first be calibrated by the *cmcalibrate* program, which performs a simulated search against random sequence to determine model-specific parameters for assigning E-values to database hits. (Rfam CM files come pre-calibrated.) The *cmsearch* program takes a calibrated CM file, searches it against a sequence database and outputs a ranked list of top scoring hits and hit alignments. The *cmalign* program takes a CM file (calibrated or not), aligns all sequences to the model and outputs a structurally annotated MSA in Stockholm format. Version 1.1 introduces the *cmscan* program for determining whether a given sequence contains homologies to any known RNA families in a CM library like Rfam. Before running *cmscan*, the CM database must be converted to a special format using *cmpress*, which enables faster scanning.

## 4 PERFORMANCE

An independent benchmark of RNA homology search (Freyhult *et al.*, 2007) found covariance model-based programs, including a previous version of Infernal, to be the most specific and sensitive of the tools tested. We present here results from an updated version of our previously published internal RMARK benchmark (Nawrocki *et al.*, 2009), mainly to indicate the relative performance of Infernal 1.1 and the previous version 1.0.2.

The RMARK3 benchmark was constructed from the seed alignments of the Rfam 10.0 database as previously described (Nawrocki *et al.*, 2009). It is composed of a set of 106 families, each represented by a training alignment of ≥5 aligned sequences and a test set of ≥1 sequences. No two test sequences are >70% identical, and no train/test sequence pair is >60% identical. The 780 test sequences were embedded into ten 1 Mb genome-like sequences, to create a benchmark ‘pseudo-genome’ of 10.16 Mb. For each included family, a model was built from the training set using the Rfam alignment, calibrated and used to search the pseudo-genome. The resulting hits from all searches were then sorted by E-value and a sensitivity versus false-positive rate ROC-like curve was generated from the results (Fig. 1).

Figure 1 shows that default Infernal 1.1 performs the benchmark searches in 0.44 h and is ∼100 times faster than the previous version 1.0.2 (49.31 h) and ∼10 000 times faster than exhaustive non-filtered 1.1 search (4359 h); yet all three search methods have similar sensitivity at the low false-positive rates necessary for large database searches. We also tested two sequence-only methods: profile HMMs implemented in HMMER3 (Eddy, 2008, 2011) and family-pairwise (Grundy, 1998) single-sequence BLASTN queries (Altschul *et al.*, 1997), which were faster (0.02 and 0.01 h, respectively), but significantly less sensitive than CMs, indicating the benefit of secondary structure modeling.

The relatively fast speed of default version 1.1 on the benchmark is maintained on real genomic sequences. The average speed is 1.5 s/Mb/query on the benchmark and 0.6 s/Mb/query on a several gigabase database that includes a sampling of 15 genomes (five each of archaea, bacteria and eukarya) using the same query models from the benchmark. As database size increases, Infernal increases filter stringency resulting in faster search rates without sacrificing appreciable sensitivity at low false-positive rates based on further RMARK benchmarking (results not shown).

Infernal is now a more practical tool for RNA homology search. The increased speed should enable its incorporation into automated sequence annotation pipelines and obviate the need for additional filtering schemes for large-scale CM searches, such as the BLAST-based filter paradigm used by Rfam (Griffiths-Jones *et al.*, 2003). Rfam-based annotation of one typical bacterial or archaeal genome (i.e. searching all 2208 Rfam 11.0 models against a 2–5 Mb target) now takes ∼1 h on a single quad-core desktop computer. Analysis of larger datasets, however, such as vertebrate genomes or all reads from a high-throughput sequencing run, still requires a compute cluster. As an example, a search of all Rfam models against the 1 Gb chicken genome would require ∼3 h on a 100-CPU compute cluster. The most expensive programs (*cmalign*, *cmcalibrate*, *cmscan* and *cmsearch*) are implemented for use with multiple threads on multi-core machines and in coarse-grained MPI versions for clusters.
